# COVID-19 pandemic shifted epidemiology for cancer screening sites: breast, cervix, colon, and rectum

**DOI:** 10.3389/fonc.2024.1481242

**Published:** 2025-01-30

**Authors:** Yevgeniy Ishkinin, Dilyara Kaidarova, Serzhan Nazarbek, Alma Zhylkaidarova, Saniya Ossikbayeva, Kamilla Mussina, Nazgul Omarbayeva

**Affiliations:** ^1^ Department of Radiation Therapy, Almaty Oncology Center, Almaty, Kazakhstan; ^2^ Kazakh Institute of Oncology and Radiology, Almaty, Kazakhstan; ^3^ Asfendiyarov Kazakh National Medical University, Almaty, Kazakhstan Asfendiyarov Kazakh National Medical University, Almaty, Kazakhstan; ^4^ Club of Experts under the Senate of Republic of Kazakhstan, Astana, Kazakhstan; ^5^ Kazakhstan Association of Healthcare Managers, Astana, Kazakhstan; ^6^ Department of Screening, Kazakh Institute of Oncology and Radiology, Almaty, Kazakhstan; ^7^ Centre for Molecular Genetic Research, Kazakh Institute of Oncology and Radiology, Almaty, Kazakhstan; ^8^ Department of Medicine, School of Medicine, Nazarbayev University, Astana, Kazakhstan; ^9^ Department of Mammology, Kazakh Institute of Oncology and Radiology, Almaty, Kazakhstan

**Keywords:** cancer screening, breast cancer, cervical cancer, colon cancer, rectum cancer, COVID-19, early-onset cancer

## Abstract

**Background:**

This study aimed to assess the epidemiological changes in breast, cervical, colon, and rectal cancers in Kazakhstan before and during COVID-19, including early-onset cancer (EOC) diagnosed between the ages of 20 and 49, using data from the oncological service of the Republic of Kazakhstan for the 2017–2022 period.

**Methods:**

The cohort comprised patients aged 20 to 49 years (EOC) and 50 years and older [late-onset cancer (LOC)] from the total number of patients diagnosed each year during the study period of 2017 to 2022 for breast, cervical, colon, or rectal cancer. In order to indicate a difference in one-time intervals and characterize the global trend over the entire study period, annual percentage change (APC) and average APC (AAPC) were calculated, respectively.

**Results:**

Breast cancer detection rates increased by 22.8% for EOC and 15.9% for LOC from 2017 to 2022, and AAPC increased by 4.3% for EOC and 3.6% for LOC. During the COVID-19 restriction period, breast cancer detection rates decreased by 6.1% for EOC and 15.6% for LOC. Cervical cancer detection rates increased by 2.3% for EOC and 7.5% for LOC from 2017 to 2022, and AAPC increased by 0.9% for EOC and 1.6% for LOC. During the COVID-19 restriction period, cervical cancer detection rates decreased by 11.3% for EOC and 3.1% for LOC. Colon cancer detection rates increased by 18.4% for EOC and 14.3% for LOC from 2017 to 2022, and AAPC increased by 3.7% for EOC and 2.9% for LOC. During the COVID-19 restriction period, colon cancer detection rates decreased by 14.4% for EOC and 5.8% for LOC. Rectal cancer detection rates increased by 13.6% for EOC and 19.2% for LOC from 2017 to 2022, and AAPC increased by 3.0% for EOC and by 3.9% for LOC. During the COVID-19 restriction period, rectal cancer detection rates increased by 18.6% for EOC and decreased by 12.0% for LOC.

**Conclusion:**

The epidemiological indicators of population cancer screening worsened during the COVID pandemic; the detection rate decreased by 6.1% for breast EOC and 11.3% for cervical EOC, while there was an increase by 38.0% in EOC for colon cancer in men and by 8.0% in EOC for rectal cancer in men and 31.1% in women.

## 1 Introduction

At the end of 2019, China informed the World Health Organization (WHO) that severe acute respiratory syndrome coronavirus 2 (SARS-CoV-2) is the virus that causes COVID-19 (1). This is a highly contagious infectious disease with contact-household transmission mechanisms, which damages the lung tissue induced by a new strain of the virus from the genus *Coronavirus* SARS CoV-2 (2). Highly contagious COVID-19 has affected the healthcare system and patients with certain preexisting medical conditions including cancer. Due to the fast-growing spread and high infection rates, lockdowns and restrictions were performed, which affected the detection and diagnosis of cancer. Therefore, cancer screening programs were suspended in many countries, and preventive care services including cancer screening programs showed a gradual decline worldwide (3–5).

Breast cancer is the most widespread cancer type worldwide. In 2020, 2.3 million new cases were diagnosed and 685,000 deaths were registered around the world (5). Early screening and detection can reduce mortality by up to 65% among breast cancer patients (5). Screening models vary in different countries. Beginning in 2008, breast cancer screening became mandatory in Kazakhstan for women at the ages of 50, 52, 54, 56, 58, and 60 until 2017. Since 2018, all women between the ages of 40 and 70 years with an interval of 2 years are subject to mandatory screening. For the screening test, the target population receives two projections of mammography of each breast. Kazakhstan implemented screening by international standards in 2011, including double reading, interpretation according to the Breast Imaging-Reporting and Data System (BI-RADS), and test result (mammogram) archiving (6).

The COVID-19 pandemic also led to a reduction in cervical cancer screening and population-based prevention activities including human papillomavirus (HPV) vaccinations. Studies have reported that HPV vaccinations declined by >70% in March 2020, and cervical cancer screening dropped by 94% due to the pandemic restrictions (7). In Kazakhstan, a major concern regarding smears according to the Bethesda System was introduced in 2011. The liquid-based cytology method was gradually introduced in 2012. Both methods are currently used for the screening test for the women age group 30–70 years with an interval of one time in 4 years. During the COVID-19 pandemic, our healthcare system also faced delays in care and cervical cancer screening tests, which led to disruptions in cancer prevention systems.

Colorectal cancer is considered the second cause of cancer deaths in the world with a mortality rate of 9.4% (8). During the COVID-19 pandemic, in 2020, more than 1.9 million new cases and 935,000 deaths occurred due to this type of cancer, which shows the significance of early screening in order to improve the mortality rate (8). Colorectal cancer screening was introduced in Kazakhstan in 2011 following the Health Development State Program of the Republic of Kazakhstan. The target population group included both sexes aged 50 to 60 years until 2017 and was expanded to 70 years in 2018, with the screening offered every 2 years. The detection of hidden blood in the stool has been used as a primary test type; however, beginning in 2013, an immunochemical (immunofluorescence) test has been used, and the in-depth diagnosis includes total colonoscopy (9). COVID-19 had a significant impact on the diagnosis of new cases of colorectal cancer and delayed onset of treatment and is significantly associated with increased mortality rates.

In 2020–2021, the COVID-19 pandemic changed approaches in the diagnostics and cancer care continuum. Fear and anxiety caused by the COVID-19 pandemic from patients and healthcare workers delayed screening for breast, cervical, and colorectal cancers, which led to diagnoses at a later stage with poorer prognoses (10). Screening rates for breast, cervical, and colon cancers were slightly below baselines, and cancer cases were lower than expected (11). Moreover, the perception of the existing barriers to screening procedures among the population worsened during COVID-19, and the disparity has grown (12, 13).

Although healthcare workers around the world adapted to meet the needs, data indicating screening availability in Kazakhstan during the quarantine are lacking (14). To our knowledge, this is the first study to describe the incidence and mortality rates of breast, cervical, and colorectal cancers during the pandemic period in Kazakhstan, even in Central Asia, using big population register data. Therefore, this study aimed to assess the epidemiological situation for cancer screening sites among the population of Kazakhstan before and during the COVID-19 pandemic. Notably, we aimed to analyze epidemiological changes in breast, cervical, and colorectal cancers in Kazakhstan, including early-onset cancer diagnosed between the ages of 20 and 49 years using data from the oncological service of the Republic of Kazakhstan for the 2017–2022 period.

## 2 Materials and methods

### 2.1 Study design and population

This is a retrospective study. The data were retrieved from statistical and analytical materials of the oncological service of the Republic of Kazakhstan for the 2017–2022 timeframe (15–20). The study population comprised patients aged 20 to 49 years [early-onset cancer (EOC)] and 50 years and older [late-onset cancer (LOC)] from the total number of patients diagnosed each year during the study period for breast, cervical, colon, and rectal cancers. There were four screening locations analyzed: breast and cervix for women and the two colorectal cancer (CRC) localizations, colon (the ascending colon, transverse colon, descending colon, and caecum) and rectum (the rectosigmoid junction, rectum, and anal canal), for women and men together and separately.

### 2.2 Statistical analyses

For the intent of this study, several indicators from the Kazakhstan Cancer Registry were calculated, and standardized indicators were analyzed according to the WHO World Standard (21). Categorical variables were described as frequencies and percentages, and continuous variables were expressed as averages. Selected indicators included standardized incidence per 100,000 population, standardized mortality per 100,000 population, mortality-to-incidence ratio in %, 1-year mortality as a percentage, mortality among the contingent of patients registered and under supervision at the end of the corresponding year, 5-year patient survival as a percentage, population and distribution by stages of newly diagnosed cancers, and early/advance stage ratio. These indicators were separately analyzed for every year in the period 2017–2022. The age-based detection study included all cancer patients registered in the oncological registry in Kazakhstan, according to data from official analytical reports of the oncological centers and “Form No. 7” and registration of patients diagnosed for the first time in their lives “Form No. 90” from 2017 to 2022. Annual percentage change (APC) was calculated by taking the difference in incidence between 1 year and the next by calculating the corresponding percentage. The average APC (AAPC) was obtained by adding all the APCs and dividing by the total number of years of the study period. APC indicates a difference in one-time intervals, whereas AAPC characterizes the global trend over the entire study period. Statistical analysis was performed using the Stata MP2 16.1 version. To determine the difference between one categorical variable and another continuous variable, analysis of variance (ANOVA) was used. *p*-Values are two-sided and reported as statistically significant at <0.05 for all analyses.

### 2.3 Ethics approval

The Ethics Committee at JSC “Kazakh Research Institute of Oncology and Radiology” (protocol No. 4-2021) approved this project. The study was performed according to both international and local ethics guidelines and regulations.

## 3 Results

### 3.1 Breast cancer

**Figure 1:**
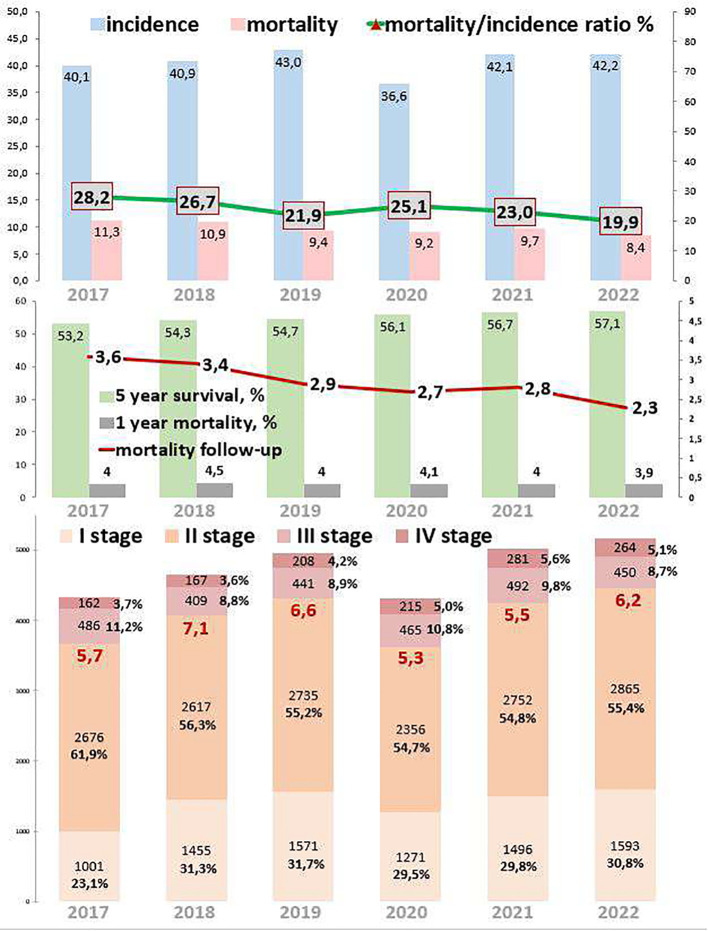
Key epidemiological indicators for breast cancer in Kazakhstan for 2017-2022.

**Figure 1** depicts the epidemiological data of the breast cancer cohort. The incidence increased by 5.2% from 2017 to 2022. During the COVID-19 restrictions, the incidence dropped to 36.6 per 100,000 in 2020, which was 14.9% lower than in 2019 and 15.0% lower than in 2021. Mortality decreased by 25.7% from 2017 to 2022. During the COVID-19 restrictions, the mortality rate was 9.2 per 100,000 in 2020, which was 3.2% higher than that from 2019 to 2021. The ratio of mortality to incidence decreased by 8.3, which was 29.4% lower than that from 2017 to 2022. During the COVID-19 restrictions, this ratio was 25.1, which was 14.6% higher than in 2019 and 8.4% higher than in 2021. One-year mortality decreased by 0.1, which was 2.5% lower than that from 2017 to 2022. During the COVID-19 restrictions, 1-year mortality was 4.1%. Mortality follow-up decreased by 2.3%, which was 36.1% lower than that from 2017 to 2022. During the COVID-19 restrictions, mortality follow-up was 2.7%. The 5-year survival rate increased by 3.9% (growth rate was 7.3%), from 53.2% in 2017 to 57.1% in 2022.

Out of 121,769 breast cancer patients, 26,485 (21.7%) had EOC while 93,958 (77.2%) had LOC, and 1,326 (1.1%) patients were aged 0–19 years. Comparing 2022 to 2017, the new cases increased by 22.8% for EOC and 15.9% for LOC. The highest APC for EOC was 8.7% in 2018, and for LOC, it was 19.8% in 2021. The AAPC increased by 4.3% for EOC and 3.6% for LOC. During the COVID-19 restriction period, breast cancer detection rates decreased by 6.1% for EOC and 15.6% for LOC (**Tables 1**, **2**).

**Table 1:** Incidence of breast, cervical, colon, and rectal cancers by EOC (20–49 years) and LOC (≥50 years) in Kazakhstan between 2017 and 2022.

	EOC	LOC	Total	*p*-Value
Breast cancer by years (%)				
2017	4,231 (21.3)	15,417 (77.6)	19,860	0.0038
2018	4,388 (21.9)	15,419 (77.0)	20,013	0.005
2019	4,445 (21.7)	15,855 (77.5)	20,448	0.0053
2020	4,124 (22.5)	13,962 (76.2)	18,332	0.0094
2021	4,490 (21.7)	15,994 (77.2)	20,727	0.0072
2022	4,807 (21.5)	17,311 (77.3)	22,389	0.0053
Cervical cancer by years (%)				
2017	897 (48.7)	945 (51.3)	1,842	0.8637
2018	874 (47.7)	958 (52.3)	1,832	0.9291
2019	852 (47.4)	945 (52.6)	1,797	0.9491
2020	756 (45.2)	916 (54.8)	1,672	0.901
2021	787 (43.6)	1,017 (56.4)	1,804	0.7896
2022	915 (47.4)	1,016 (52.6)	1,931	0.9499
Colon cancer by years (%)				
2017	174 (10.3)	1,513 (89.7)	1,687	0.0014
2018	167 (10.0)	1,494 (89.6)	1,667	0.0005
2019	167 (9.8)	1,543 (90.1)	1,712	0.0005
2020	191 (11.6)	1,453 (88.3)	1,646	0.0009
2021	188 (11.2)	1,496 (88.7)	1,686	0.002
2022	206 (11.2)	1,629 (88.5)	1,840	0.0007
Rectal cancer by years (%)				
2017	176 (12.2)	1,268 (87.8)	1,444	0.0007
2018	176 (11.3)	1,374 (88.6)	1,551	0.001
2019	161 (10.0)	1,455 (90.0)	1,617	0.001
2020	191 (13.0)	1,280 (87.0)	1,471	0.0028
2021	188 (11.7)	1,414 (88.2)	1,604	0.0024
2022	200 (11.7)	1,512 (88.3)	1,713	0.0037

EOC, early-onset cancer; LOC, late-onset cancer.

**Table 2:** Incidence by age for breast cancer in Kazakhstan between 2017 and 2022.

Ages	2017	2018	2019	2020	2021	2022	2017–2022
0–19 years	212 (1.1%)	206 (1.0%)	148 (0.7%)	246 (1.3%)	243 (1.2%)	271 (1.2%)	1,326 (1.1%)
20–24 years	132 (0.7%)	137 (0.7%)	109 (0.5%)	108 (0.6%)	100 (0.5%)	113 (0.5%)	699 (0.6%)
25–28 years	236 (1.2%)	219 (1.1%)	166 (0.8%)	162 (0.9%)	164 (0.8%)	180 (0.8%)	1,127 (0.9%)
29–34 years	599 (3.0%)	600 (3.0%)	595 (2.9%)	556 (3.0%)	615 (3.0%)	642 (2.9%)	3,607 (3.0%)
35–39 years	693 (3.5%)	726 (3.6%)	789 (3.9%)	766 (4.2%)	796 (3.8%)	950 (4.2%)	4,720 (3.9%)
40–44 years	1,097 (5.5%)	1,131 (5.7%)	1,237 (6.0%)	1,108 (6.0%)	1,256 (6.1%)	1,248 (5.6%)	7,077 (5.8%)
45–49 years	1,474 (7.4%)	1,575 (7.9%)	1,549 (7.6%)	1,424 (7.8%)	1,559 (7.5%)	1,674 (7.5%)	9,255 (7.6%)
50–54 years	2,037 (10.3%)	1,921 (9.6%)	1,854 (9.1%)	1,706 (9.3%)	1,949 (9.4%)	2,006 (9.0%)	11,473 (9.4%)
55–59 years	2,716 (13.7%)	2,580 (12.9%)	2,638 (12.9%)	2,264 (12.3%)	2,456 (11.8%)	2,605 (11.6%)	15,259 (12.5%)
60–64 years	2,730 (13.7%)	2,802 (14.0%)	3,018 (14.8%)	2,779 (15.0%)	3,240 (15.6%)	3,411 (15.2%)	17,980 (14.8%)
65–69 years	2,726 (13.7%)	2,987 (14.9%)	3,066 (15.0%)	2,689 (14.7%)	2,929 (14.1%)	3,194 (14.3%)	17,591 (14.4%)
70–74 years	1,547 (7.8%)	1,678 (8.4%)	1,950 (9.5%)	1,989 (10.8%)	2,542 (12.3%)	2,751 (12.3%)	12,457 (10.2%)
75–79 years	2,166 (10.9%)	1,850 (9.2%)	1,561 (7.6%)	1,049 (5.7%)	1,103 (5.3%)	1,345 (6.0%)	9,074 (7.5%)
80–84 years	996 (5.0%)	1,139 (5.7%)	1,324 (6.5%)	1,108 (6.0%)	1,272 (6.1%)	1,400 (6.3%)	7,239 (5.9%)
85 years and older	499 (2.5%)	462 (2.3%)	444 (2.2%)	378 (2.1%)	503 (2.4%)	599 (2.7%)	2,885 (2.4%)
EOC 20–49 years	4,231 (21.3%)	4,388 (21.9%)	4,445 (21.7%)	4,124 (22.5%)	4,490 (21.7%)	4,807 (21.5%)	26,485 (21.8%)
LOC over 50 years	15,417 (77.6%)	15,419 (77.0%)	15,855 (77.5%)	13,962 (76.2%)	15,994 (77.2%)	17,311 (77.3%)	93,958 (77.2%)
Total	19,860	20,013	20,448	18,332	20,727	22,389	121,769

EOC, early-onset cancer; LOC, late-onset cancer.

### 3.2 Cervical cancer

**Figure 2:**
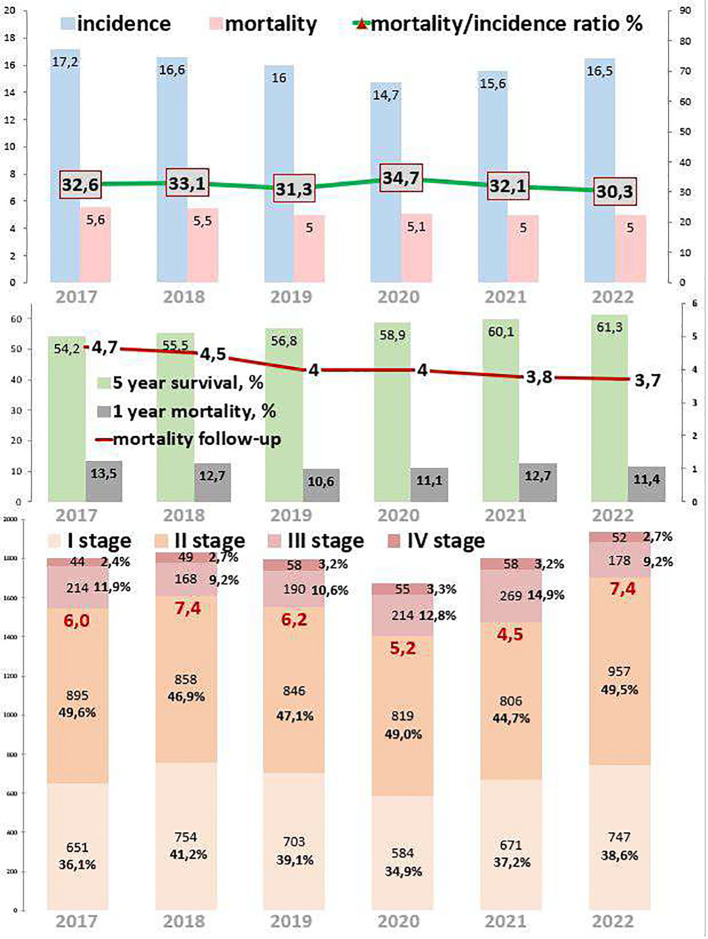
Key epidemiological indicators for cervical cancer in Kazakhstan from 2017 to 2022.

**Figure 2** describes cervical cancer epidemiological trends. The incidence decreased by 4.1% from 2017 to 2022. During the COVID-19 restrictions, the incidence dropped to 14.7 in 2020, which is 14.5% lower than in 2019 and 6.1% lower than in 2021. Mortality decreased by 10.7% from 2017 to 2022. During the COVID-19 restrictions, mortality was 5.1 in 2020, and it remained stable from 2019 to 2021. The ratio of mortality to incidence decreased by 2.3%, which was 20.6% lower than that from 2017 to 2022. During the COVID-19 restrictions, this ratio was 34.7, which was 10.9% higher than in 2019 and 7.5% higher than in 2021. One-year mortality decreased by 2.1%, which was 15.6% lower than that from 2017 to 2022. During the COVID-19 restrictions, 1-year mortality was 11.1%. Mortality follow-up decreased by 1.0%, which was 21.3% lower than that from 2017 to 2022. During the COVID-19 restrictions, mortality follow-up was 4.0%. The 5-year survival increased by 7.1% (growth rate by 17.0%), from 52.4% in 2017 to 61.3% in 2022.

Out of 10,878 cervical cancer patients, 5,081 (46.7%) had EOC, while 5,797 (53.3%) had LOC. New cases of cervical cancer increased by 5.0% over the study period in both age groups. Comparing 2022 to 2017, the new cases increased by 2.3% for EOC and 7.5% for LOC. The highest APC for EOC was 16.6% in 2022, and for LOC, it was 11.0% in 2021. The AAPC increased by 0.9% for EOC and 1.6% for LOC. During the COVID-19 restriction period, cervical cancer detection rates decreased by 11.3% for EOC and 3.1% for LOC (**Tables 1**, **3**). However, there was no statistically significant difference in the incidence of cervical cancer for the period of 2017 and 2022.

**Table 3:** Incidence by age for cervical cancer in Kazakhstan for 2017–2022.

Ages	2017	2018	2019	2020	2021	2022	2017–2022
0–19 years	0	0	0	0	0	0	0
20–24 years	8 (0.4%)	9 (0.5%)	7 (0.4%)	3 (0.2%)	3 (0.2%)	4 (0.2%)	34 (0.3%)
25–28 years	34 (1.8%)	36 (2.0%)	18 (1.0%)	23 (1.4%)	17 (0.9%)	28 (1.5%)	156 (1.4%)
29–34 years	124 (6.7%)	130 (7.1%)	135 (7.5%)	96 (5.7%)	123 (6.8%)	146 (7.6%)	754 (6.9%)
35–39 years	181 (9.8%)	163 (8.9%)	178 (9.9%)	162 (9.7%)	187 (10.4%)	209 (10.8%)	1,080 (9.9%)
40–44 years	276 (15.0%)	245 (13.4%)	261 (14.5%)	232 (13.9%)	220 (12.2%)	247 (12.8%)	1,481 (13.6%)
45–49 years	274 (14.9%)	291 (15.9%)	253 (14.1%)	240 (14.4%)	237 (13.1%)	281 (14.6%)	1,576 (14.5%)
50–54 years	269 (14.6%)	269 (14.7%)	249 (13.9%)	233 (13.9%)	259 (14.4%)	251 (13.0%)	1,530 (14.1%)
55–59 years	249 (13.5%)	249 (13.6%)	241 (13.4%)	208 (12.4%)	243 (13.5%)	248 (12.8%)	1,438 (13.2%)
60–64 years	169 (9.2%)	177 (9.7%)	186 (10.4%)	207 (12.4%)	215 (13.5%)	230 (12.8%)	1,184 (13.2%)
65–69 years	127 (6.9%)	127 (6.9%)	143 (8.0%)	139 (8.3%)	140 (7.8%)	142 (7.4%)	818 (7.5%)
70–74 years	48 (2.6%)	58 (3.2%)	59 (3.3%)	73 (4.4%)	104 (5.8%)	82 (4.2%)	424 (3.9%)
75–79 years	53 (2.9%)	44 (2.4%)	33 (1.8%)	21 (1.3%)	28 (1.6%)	30 (1.6%)	209 (1.9%)
80–84 years	24 (1.3%)	28 (1.5%)	29 (1.6%)	31 (1.9%)	24 (1.3%)	24 (1.2%)	160 (1.5%)
85 years and older	6 (0.3%)	6 (0.3%)	5 (0.3%)	4 (0.2%)	4 (0.2%)	9 (0.5%)	34 (0.3%)
EOC 20–49 years	897 (48.7%)	874 (47.7%)	852 (47.4%)	756 (45.2%)	787 (43.6%)	915 (47.4%)	5,081 (46.7%)
LOC over 50 years	945 (51.3%)	958 (52.3%)	945 (52.6%)	916 (54.8%)	1,017 (56.4%)	1,016 (52.6%)	5,797 (53.3%)
Total	1,842	1,832	1,797	1,672	1,804	1,931	10,878

EOC, early-onset cancer; LOC, late-onset cancer.

### 3.3 Colon cancer

**Figure 3:**
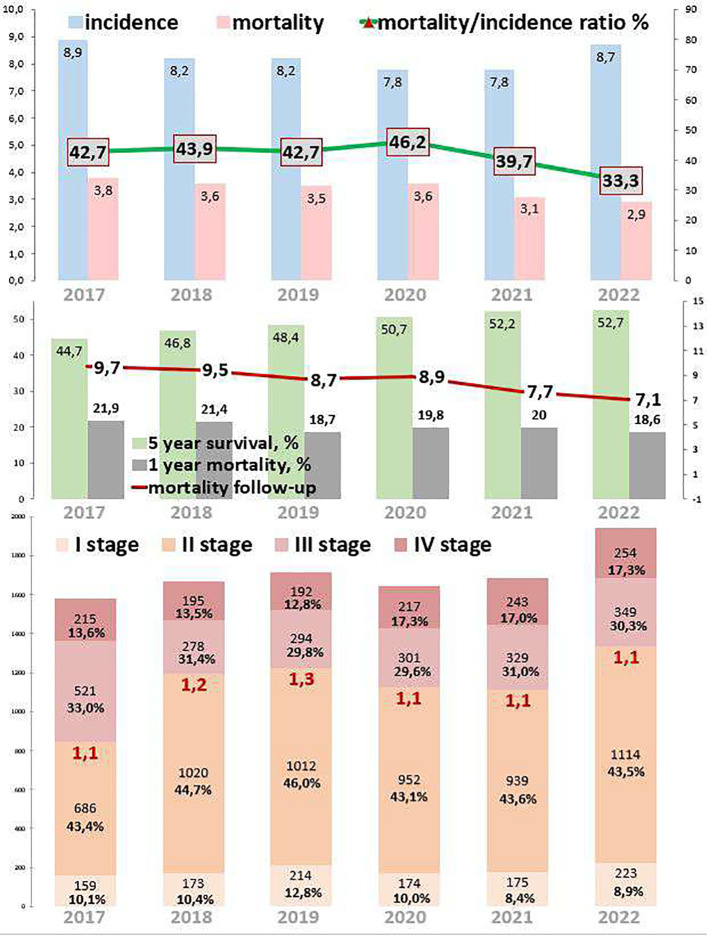
Key epidemiological indicators for colon cancer in Kazakhstan from 2017 to 2022.

Colon cancer epidemiological trends are presented in **Figure 3**. The incidence decreased by 2.2% from 2017 to 2022. During the COVID-19 restrictions, the incidence was 7.8 cases per 100,000 in 2020, which was 4.9% lower than in 2019 and the same as in 2021. Mortality decreased by 23.7% from 2017 to 2022. During the COVID-19 restrictions, mortality was 3.6 cases per 100,000 in 2020, which was 2.9% higher than in 2019 and 13.9% higher than in 2021. The ratio of mortality to incidence decreased to 9.4, which was 22.0% lower than that from 2017 to 2022. During the COVID-19 restrictions, this ratio was 46.2, which was 8.2% higher than in 2019 and 14.1% higher than in 2021.

Out of 10,238 colon cancer patients, 1,093 (10.7%) had EOC, while 9,128 (89.2%) had LOC, and 17 (0.2%) patients were aged 0–19 years (**Tables 1**, **4**). Comparing 2022 to 2017, new cases increased by 18.4% for EOC and by 14.3% for LOC. The highest APC for EOC was 14.4% in 2020, and for LOC, it was 15.6% in 2022. The AAPC increased by 3.7% for EOC and by 2.9% for LOC. During the COVID-19 restriction period, colon cancer detection rates increased by 14.4% for EOC and decreased by 5.8% for LOC.

**Table 4:** Incidence by age for colon cancer of both sexes in Kazakhstan for 2017–2022.

Ages	2017	2018	2019	2020	2021	2022	2017–2022
0-19 years	0	6 (0.4%)	2 (0.1%)	2 (0.1%)	2 (0.1%)	5 (0.3%)	17 (0.2%)
20-24 years	3 (0.2%)	2 (0.1%)	3 (0.2%)	11 (0.7%)	5 (0.3%)	4 (0.2%)	28 (0.3%)
25-28 years	5 (0.3%)	5 (0.3%)	4 (0.2%)	5 (0.3%)	2 (0.1%)	6 (0.3%)	27 (0.3%)
29-34 years	11 (0.6%)	24 (1.3%)	24 (1.3%)	28 (1.7%)	17 (0.9%)	21 (1.1%)	125 (1.2%)
35-39 years	34 (1.8%)	29 (1.6%)	30 (1.7%)	26 (1.6%)	33 (1.8%)	35 (1.8%)	187 (1.8%)
40-44 years	48 (2.6%)	47 (2.6%)	45 (2.5%)	44 (2.6%)	49 (2.7%)	51 (2.6%)	284 (2.8%)
45-49 years	73 (4.0%)	60 (3.3%)	61 (3.4%)	77 (4.6%)	82 (4.5%)	89 (4.6%)	442 (4.3%)
50-54 years	110 (6.0%)	120 (6.6%)	127 (7.1%)	121 (7.2%)	118 (6.5%)	143 (7.4%)	739 (7.2%)
55-59 years	223 (12.1%)	231 (12.6%)	221 (12.3%)	201 (12.0%)	180 (10.0%)	207 (10.7%)	1,263 (12.3%)
60-64 years	275 (14.9%)	268 (14.6%	297 (16.5%)	286 (17.1%)	310 (17.2%)	346 (17.9%)	1,782 (17.4%)
65-69 years	355 (19.3%)	311 (17.0%)	323 (18.0%)	301 (18.0%)	332 (18.4%)	245 (12.7%)	1,867 (18.2%)
70-74 years	175 (9.5%)	174 (9.5%)	230 (12.8%)	234 (14.0%)	275 (15.2%)	324 (16.8%)	1,412 (13.8%)
75-79 years	236 (12.8%)	228 (12.4%)	177 (9.8%)	152 (9.1%)	123 (6.8%)	166 (8.6%)	1,082 (10.6%)
80-84 years	101 (5.5%)	129 (7.0%)	130 (7.2%)	127 (7.6%)	124 (6.9%)	150 (7.8%)	761 (7.4%)
85 years and older	38 (2.1%)	33 (1.8%)	38 (2.1%)	31 (1.9%)	34 (1.9%)	48 (2.5%)	222 (2.2%)
EOC 20-49 years	174 (10.3%)	167 (10.0%)	167 (9.8%)	191 (11.6%)	188 (11.2%)	206 (11.2%)	1,093 (10.7%)
LOC over 50 years	1,513 (89.7%)	1,494 (89.6%)	1,543 (90.1%)	1,453 (88.3%)	1,496 (88.7%)	1,629 (88.5%)	9,128 (89.2%)
Total	1,687	1,667	1,712	1,646	1,686	1,840	10,238

EOC, early-onset cancer; LOC, late-onset cancer.

### 3.4 Rectal cancer

**Figure 4:**
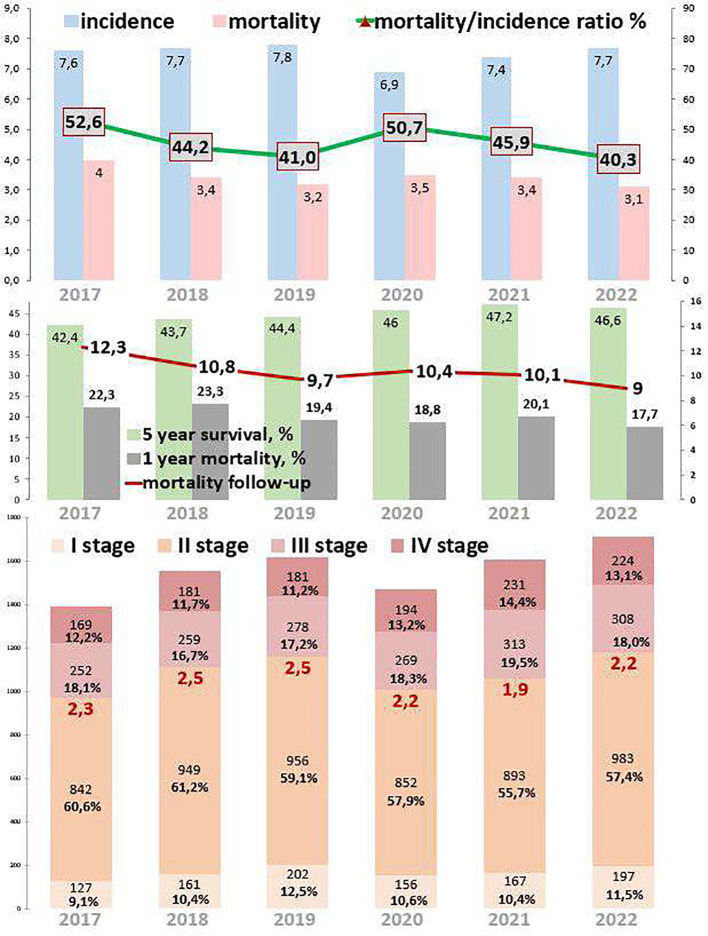
Key epidemiological indicators for rectum cancer in Kazakhstan from 2017 to 2022.

Rectal cancer epidemiological trends are represented in **Figure 4**. The incidence of rectal cancer increased by 2.2% from 2017 to 2022. During the COVID-19 restrictions, the incidence dropped to 6.9 cases per 100,000 in 2020, which was 11.5% lower than that in 2019 and 7.2% lower than that in 2021. The mortality decreased by 22.5% from 2017 to 2022. However, during the COVID-19 restrictions, mortality increased to 3.5 cases in 2020, which was 9.4% higher than that in 2019 and 2.9% higher than that in 2021. The ratio of mortality to incidence decreased by 12.3, which was 23.4% lower than that from 2017 to 2022. During the COVID-19 restrictions, this ratio increased to 50.7, which was 23.7% higher than that in 2019 and 9.5% higher than that in 2021. One-year mortality decreased by 4.6%, which was 20.6% lower than that from 2017 to 2022. During the COVID-19 restrictions, 1-year mortality was 18.8%. Mortality follow-up decreased by 3.3%, which was 26.8% lower than that from 2017 to 2022. However, during the COVID-19 restrictions, mortality follow-up increased to 10.4%. The 5-year survival increased by 4.2% (growth rate by 9.9%), from 44.7% in 2017 to 52.7% in 2022.

Out of 9,400 rectal cancer patients, 1,092 (11.6%) had EOC, while 8,303 (88.3%) had LOC, and five (0.1%) patients were aged 0–19 years (**Tables 1**, **5**). Comparing 2022 to 2017, new cases increased by 13.6% for EOC and by 19.2% for LOC. The most significant APC for EOC was 18.6% in 2020, and for LOC, it was 10.5% in 2021. The AAPC increased by 3.0% for EOC and by 3.9% for LOC. During the COVID-19 restriction period, rectal cancer detection rates for EOC increased by 18.6%, while detection rates for LOC decreased by 12.0% (**Table 6**).

**Table 5:** Incidence by age for rectal cancer of both sexes in Kazakhstan for 2017–2022.

Ages	2017	2018	2019	2020	2021	2022	2017–2022
0–19 years	0	1 (0.1%)	1 (0.1%)	0	2 (0.1%)	1 (0.1%)	5 (0.1%)
20–24 years	5 (0.3%)	2 (0.1%)	3 (0.2%)	2 (0.1%)	3 (0.2%)	0	15 (0.2%)
25–28 years	10 (0.5%)	5 (0.3%)	1 (0.1%)	4 (0.2%)	2 (0.1%)	4 (0.2%)	26 (0.3%)
29–34 years	16 (0.9%)	14 (0.8%)	18 (1.0%)	25 (1.5%)	20 (1.1%)	15 (0.8%)	108 (1.1%)
35–39 years	25 (1.4%)	26 (1.4%)	27 (1.5%)	38 (2.3%)	30 (1.7%)	33 (1.7%)	179 (1.9%)
40–44 years	40 (2.2%)	49 (2.7%)	40 (2.2%)	47 (2.8%)	57 (3.2%)	59 (3.1%)	292 (3.1%)
45–49 years	80 (4.3%)	80 (4.4%)	72 (4.0%)	75 (4.5%)	76 (4.2%)	89 (4.6%)	472 (5.0%)
50–54 years	123 (6.7%)	135 (7.4%)	140 (7.8%)	116 (6.9%)	127 (7.0%)	148 (7.7%)	789 (8.4%)
55–59 years	194 (10.5%)	219 (12.0%)	237 (13.2%)	184 (11.0%)	223 (12.4%)	214 (11.1%)	1,271 (13.5%)
60–64 years	229 (12.4%)	279 (15.2%)	292 (16.2%)	281 (16.8%)	296 (16.4%)	359 (18.6%)	1,736 (18.5%)
65–69 years	250 (13.6%)	285 (15.6%)	314 (17.5%)	277 (16.6%)	302 (16.7%)	323 (16.7%)	1,751 (18.6%)
70–74 years	163 (8.8%)	145 (7.9%)	189 (10.5%)	202 (12.1%)	237 (13.1%)	241 (12.5%)	1,177 (12.5%)
75–79 years	200 (10.9%)	170 (9.3%)	132 (7.3%)	93 (5.6%)	96 (5.3%)	102 (5.3%)	793 (8.4%)
80–84 years	77 (4.2%)	117 (6.4%)	124 (6.9%)	102 (6.1%)	102 (5.7%)	91 (4.7%)	613 (6.5%)
85 years and older	32 (1.7%)	24 (1.3%)	27 (1.5%)	25 (1.5%)	31 (1.7%)	34 (1.8%)	173 (1.8%)
EOC 20–49 years	176 (12.2%)	176 (11.3%)	161 (10.0%)	191 (13.0%)	188 (11.7%)	200 (11.7%)	1,092 (11.6%)
LOC over 50 years	1,268 (87.8%)	1,374 (88.6%)	1,455 (90.0%)	1,280 (87.0%)	1,414 (88.2%)	1,512 (88.3%)	8,303 (88.3%)
**Total**	1,444	1,551	1,617	1,471	1,604	1,713	9,400

EOC, early-onset cancer; LOC, late-onset cancer.

**Table 6:** Summary for incidence of early- and late-onset cancer for screening of different types of cancer by gender disparities.

	Breast cancer	Cervical cancer	Colon cancer		Rectal cancer	
			Male	Female	Male	Female
Total no. of patients	121,769	10,878	4,744	5,594	4,947	4,453
Age at diagnosis between 2017 and 2022	58.0	51.6	64.1	65.3	63.4	64.2
Mean age at diagnosis in 2017	57.8	51.1	64.3	65.6	63.6	64.6
Mean age at diagnosis in 2018	58.1	51.3	64.3	65.3	63.6	64.4
Mean age at diagnosis in 2019	58.4	51.5	64.4	65.2	63.9	64.5
Mean age at diagnosis in 2020	57.5	52.2	64.0	64.6	63.2	63.6
Mean age at diagnosis in 2021	58.3	52.3	63.9	65.3	63.1	64.2
Mean age at diagnosis in 2022	58.2	51.5	63.7	65.9	63.1	64
Number of cases in dynamics	+17.2	+5.0	+15.3	+14.2	+23.2	+13.6
2017–2022 EOC, %	21.7	46.7	11.3	10.0	16.7	12
2017–2022 LOC, %	77.2	53.3	88.7	90.0	88.7	88
EOC in dynamics	+22.8	+2.3	+17.6	+19.1	+16.7	+10.5
LOC in dynamics	+15.9	+7.5	+15.0	+13.7	+24.1	+14.1
AAPC EOC	+4.3	+0.9	+4.7	+4.0	+3.4	+3.7
AAPC LOC	+3.6	+1.6	+3.0	+2.9	+4.9	+2.9
EOC at COVID-19 restriction period	−6.1	−11.3	+38	−3.1	+8	+31.1
LOC at COVID-19 restriction period	−15.6	−3.1	−4.5	−6.9	−14.7	−8.9

EOC, early-onset cancer; LOC, late-onset cancer; AAPC, average annual percentage change.

**Table 6** provides an insight summary into the trends and dynamics of cancer incidence, emphasizing the impact of COVID-19 restrictions on cancer detection rates by gender disparities.

## 4 Discussion

This is the first study to describe the incidence and mortality rates of breast, cervical, and colorectal cancers during the pandemic period in Kazakhstan, even in Central Asia, using big population register data. This is a global public health problem that requires the utmost attention to reduce cancer incidence in young patients. This study investigated overall decreased incidence and mortality rates of breast and cervical cancers and increased detection rate of colorectal cancer during the COVID-19 restriction period. These findings, which may reflect similar situations in other countries also experiencing a decline in screening and diagnostic procedures, may present the first decline in survival rates observed during the pandemic period. Routine cancer screening is a primary preventative care measure that decreased dramatically at the start of the pandemic, with a 94% decrease in both breast cancer screening and cervical cancer screening and an 86% decrease in colon cancer screening. Rates of breast and colon cancer screening remain slightly below historical baselines, down at 2.7% and 3.4%, respectively. However, cervical cancer screening rates are still 10% below historical baselines (22, 30).

According to the results of our study, the AAPC for early-onset cancer increased between 2017 and 2022. Notably, breast cancer increased by 4.3%, cervical cancer increased by 0.9%, colon cancer increased by 4.7% among men and 4.0% among women, and rectal cancer increased by 3.4% among men and 3.7% among women. Despite the increase in EOC and LOC between 2017 and 2022, during the COVID-19 restrictions, for breast cancer, the detection rate of EOC decreased by 6.1% and that for LOC decreased by 15.6%; for cervical cancer, the detection rate of EOC decreased by 11.3%, and that of LOC decreased by 3.1%. The same trend can be observed in several studies (23–27). Restrictions imposed by different countries in order to minimize the risk of being infected had a significant effect on cancer care all over the world, especially on cancer screening procedures. For instance, a study from the Netherlands revealed that restrictions resulted in a 50% decrease in the detection rate for breast cancer cases among the 50–74-year age group. In Slovenia, cervical cancer screening programs led to a 92% decline in screening, a 70% decline in follow-up visits, and a 68% decrease in HPV testing (4). Delays in screening programs, diagnosis, and treatment during the pandemic may have led to an increase in breast cancer incidence. This may have resulted in a higher proportion of LOC and an increase in excess mortality from cancer. Moreover, due to these effects, the decrease in mortality from some cancers may have slowed down or even reversed. That is why it is essential to resume cancer screening, diagnosis, and treatment procedures in order to hold cancer incidence and mortality rates at a level as it was before the pandemic period.

However, for colon and rectal cancers, quite a different trend was observed. During the COVID-19 restrictions, for colon cancer, the detection rate of EOC increased by 14.4% (for men, it increased by 38.0%, and for women, it decreased by 3.1%) and that of LOC decreased by 5.8% (for men, it decreased by 4.5%, and for women, it decreased by 6.9%); for rectal cancer, the detection rate of EOC increased by 18.6% (for men, it increased by 8.0%, and for women, it increased by 31.1%) and that of LOC decreased by 12.0% (for men, it decreased by 14.7%, and for women, it decreased by 8.9%), which is contrary for breast and cervical cancer results. Several studies have revealed increased use of fecal immunochemical test (FIT) during the COVID-19 pandemic, which is associated with an increased rate of detection of early colorectal cancer (8, 9, 13, 28). Despite the recommendation to perform colorectal cancer screening in many countries, due to the COVID-19 pandemic restrictions, cancer screening programs were disrupted for an unknown period of time, which led to a decreased detection rate of LOC of colorectal cancer cases (28, 29). Therefore, in order to compensate for this decrease and to prevent further decline in cancer screening, further recommendations need to be addressed for the government and public health workers. First, consider the symptoms of cancers seriously and refer to diagnostic centers in a timely manner. Second, replace invasive methods with non-invasive methods in cancer screening programs. Third, increase the capacity of screening centers in order to compensate for the reduction of detection rate during the restriction period. Fourth, implement free or low-cost screening programs.

This is the first study to describe incidence and mortality rates of breast, cervical, and colorectal cancers during the pandemic restriction period in Kazakhstan, even in Central Asia, using big population register data. Moreover, the study assessed the change in the epidemiological situation and its results among the population of Kazakhstan before and during COVID-19. However, there were several limitations of our study. First, there is no clear boundary about the end and impact of restrictions during the COVID-19 pandemic. The greatest restrictions began on March 13, 2020, and continued throughout 2020 with a peak in the summer of 2020. In 2021, the COVID-19 pandemic in Kazakhstan reached a new level, and new strains of the virus appeared. However, in February 2021 a vaccination campaign began, and the restrictions were already lesser than those in 2020. Since September 2021, a gradual easing of COVID-19 pandemic restrictions occurred in Kazakhstan. According to epidemiological indicators, it is clear that the greatest failure in diagnostics occurred in 2020, but some restrictions extended to 2021, which was not taken into account in this study. Officially, the end of the COVID-19 pandemic was announced only in May 2023. The second limitation of our study is that there was no database by surnames. Data from official reports of oncology centers and the oncology institute were used. Some patients could have moved from one region to another during the year, and contamination and duplication of indicators could have occurred. Unfortunately, it is impossible to calculate and exclude these data, but such cases, if they existed, could not affect the overall picture since after transfer they were put on dispensary records with the note “translocation”. The third limitation of our study is a lack of randomization and comparison with other oncological localizations for which screening examination was not performed.

## 5 Conclusion

The results of the study showed that epidemiological indicators of population cancer screening improved from 2017 to 2022 but worsened during the maximum restrictions of the COVID-19 pandemic. Despite the increase in early-onset cancer, for breast cancer during the COVID-19 restriction period, the detection rate of EOC decreased by 6.1%; for cervical cancer, EOC decreased by 11.3%, while there was an increase in EOC for colon cancer in men by 38.0% and rectal cancer in men by 8.0% and women by 31.1%. Moreover, the results of this study highlight the importance of large-scale responses that could mitigate the detrimental association between the COVID-19 pandemic and worse cancer outcomes in patients with breast, cervical, colon, or rectal cancer.

## Data Availability

The datasets presented in this article are not readily available because data collected cannot be shared outside of the research institution. Requests to access the datasets should be directed to corresponding author. Requests to access the datasets should be directed to https://drive.google.com/file/d/1lXye8lkJRg7G8Tn96gjGoI3brv4buv6e/view, https://drive.google.com/drive/folders/1S6EnWQcSF_6qiurff1uGipcf2Mq98FnR.
